# Phytochemicals as Modulators of PPARs and RXRs

**DOI:** 10.1155/2010/407650

**Published:** 2011-05-02

**Authors:** Joshua K. Ko, Susanna S. Lee, Harry Martin

**Affiliations:** ^1^Center for Cancer and Inflammation Research, School of Chinese Medicine, Hong Kong Baptist University, Kowloon, Hong Kong; ^2^School of Life Sciences, The Chinese University of Hong Kong, Shatin, NT, Hong Kong; ^3^The New Zealand Institute for Plant & Food Research Limited, Palmerston North, New Zealand

Welcome to this special issue of PPAR Research on “Phytochemicals as modulators of PPARs and RXRs.” Plants have played a crucial role in the prevention and treatment of human diseases for thousands of years. A significant number of contemporary drugs with high efficacy are derived from herbal origin. These natural phytochemicals, including certain nutrients, are therefore of great medicinal value.

Peroxisome proliferator-activated receptors (PPARs) belong to the steroid hormone receptor superfamily that bind to and are activated by fatty acids, eicosanoids, and a group of xenobiotics called peroxisome proliferators. Each of these receptors binds to specific peroxisome proliferator response elements (PPRE) as a heterodimer with a retinoid X receptor (RXR). Binding to these receptors has been proven to control the pathological conditions associated with obesity, aging-related diseases, inflammation, immune disorder, cell cycle regulation as well as cancer. There is a strong mechanistic basis for PPAR and RXR targeting, as these nuclear receptors are transcriptional factors that modulate gene expression relevant to the control of blood glucose and lipids, as well as the processes of inflammation and carcinogenesis. Since PPARs play an important role in lipid metabolism, the search for natural ligands had begun with fatty acids and eicosanoids. In fact, the discovery that many natural dietary compounds, especially phytochemicals, are PPAR activators is of great significance for human health. With metabolic syndrome, diabetes, and chronic systemic inflammatory diseases now reaching pandemic proportions, the importance of the discovery of natural PPAR ligands is self-evident.

The special issue begins with a review of terpenoids as modulators of the PPAR family. To date more than 40,000 terpenoids have been described. Also known as isoprenoids, terpenoids constitute one of the largest and most diverse group of phytochemicals. Members of this phytochemical family are well known to us as the flavours of cinnamon, ginger, and cloves and the scents of camphor and eucalyptus. The vitamin A precursors known as carotenoids and psychoactive cannabinoids are also forms of terpenoid. In this issue, T. Goto and colleagues review the biosynthesis, metabolism, and plant origins of various dietary terpenoids. The physiological effects of these compounds on carbohydrate and lipid metabolism are described in terms of their PPAR*γ* and PPAR*α* activating abilities. This is followed by the research article on the kinetic assessment and therapeutic modulation of metabolic and obesity-associated inflammatory profiles by a high-fat diet, whereas the whole process was found to be mediated by PPAR activation. Another research article has proposed glycyrrhizic acid, the bioactive compound extracted from roots of licorice (*Glycyrrhiza glabra*), as a potent PPAR*γ* agonist. This phytochemical is capable of ameliorating metabolic syndrome by affecting glucose homeostasis, lipid metabolism, and adipogenesis. The focus is then shifted to the findings on verbascoside, a phytochemical that exerts a strong anti-inflammatory activity against the development of experimental inflammatory bowel disease. This work by the S. Cuzzocrea team has proven that verbascoside is a PPAR*α* ligand by using a knock-out animal model. S. N. Lewis and colleagues conclude this special issue with a comprehensive review of computational approaches to the discovery of phytochemical ligands for PPAR*α* and PPAR*γ*. This timely review is an excellent reference document for the existing 3D structures of PPAR receptors cocrystallized with a variety of ligands and available on-line at the RCSB database. Lewis gives an expert description of the potential for *in silico* screening of phytochemical databases. The challenges of applying computational techniques to the analysis of a flexible ligand binding to a flexible receptor are described. This review is a most helpful exposition of the state-of-the-art computational methods regarding natural PPAR ligand discovery.

*Joshua K. Ko* *Joshua K. Ko* *Susanna S. Lee* *Susanna S. Lee* *Harry Martin* *Harry Martin*

